# The Cost of Transferring Dialysis Care From the Employer-Based Market to Medicare

**DOI:** 10.1001/jamanetworkopen.2021.2113

**Published:** 2021-03-18

**Authors:** Eugene Lin

**Affiliations:** 1 Division of Nephrology, Department of Medicine, University of Southern California, Los Angeles; 2 Leonard D Schaeffer Center for Health Policy and Economics, University of Southern California, Los Angeles; 3 University Kidney Research Organization, Kidney Research Center, Los Angeles, California

## Abstract

This cohort study investigates the frequency and characteristics of patients with end-stage kidney disease receiving dialysis who prematurely transition from their employer-based group health plan to Medicare.

## Introduction

Many patients with end-stage kidney disease (ESKD) retain their employer-based group health plan (EGHP) when they start dialysis.^1^ Patients with EGHPs may obtain Medicare as a secondary payer, but the EGHP remains the primary payer for 30 months during what is known as a coordination period.^2^ If patients prematurely drop their EGHPs (eg, if they become unemployed or if their employers or payers stop offering favorable plans), Medicare becomes the primary payer, which frees the EGHPs of their financial obligations. This cohort study investigated the frequency of premature switches, the characteristics of patients switching early, and the resulting Medicare spending.

## Methods

The University of Southern California’s institutional review board approved this study. The US Renal Data System (USRDS) was used to identify all US adults (aged 62 years or younger) who had EGHP coverage when their dialysis for ESKD began between January 1, 2007, and December 31, 2014 (eAppendix in the Supplement). Patients were observed through the coordination period or death, with the latest follow-up being October 31, 2017. The USRDS is a deidentified registry of administrative data already collected by the Centers for Medicare & Medicaid Services, and thus patients did not provide informed consent. Analyses were conducted from June 2020 to January 2021 and followed the Strengthening the Reporting of Observational Studies in Epidemiology (STROBE) reporting guideline.

Premature switches to Medicare (ie, before the end of the EGHP coordination period) were identified, and the extra months of Medicare use were totaled. Patients were stratified into hospitalization probability quartiles to proxy for severity of illness. To estimate hospitalization probabilities, regression coefficients were applied from a multivariable logistic regression in adults aged 62 or younger who started dialysis with fee-for-service Medicare as the primary payer. The dependent variable was whether the patient was hospitalized in a 12-month period, and independent variables were patient characteristics and comorbidities, facility characteristics, and zip code sociodemographics (eTable in the Supplement).

A Cox proportional hazards model was used to estimate the likelihood of premature switching by hospitalization probability quartile. Ordinary least squares were used to compare total Medicare spending (as the primary or secondary payer) when switching prematurely, switching at the coordination period, and switching after the coordination period or never. Statistical significance was set at *P* = .05, and tests were 2-sided. Statistical analysis was performed using SAS version 9.4 (SAS Institute) and Stata version 14.0, MP edition (StataCorp).

## Results

A total of 113 693 US adults aged 62 years or younger (mean [SD] age, 50 [10] years; 69 357 [61%] men) who started dialysis with an EGHP were included in this study and followed for a mean of 789 (309) days (maximum of 1007 days). The study demographic characteristics included 71 406 (63%) who were White patients, 35 048 (31%) Black patients, 7239 (2%) other, which included Asian patients, Native American patients, or other patients in the database, and 14 511 (13%) Hispanic patients. Patients who switched from EGHP to Medicare prematurely (37 696 [33%]) contributed to 711 528 additional months of Medicare (mean [SD], 19 [12] additional months per patient who switched early) (Table 1). Patients with a higher hospitalization risk were more likely to switch from their EGHP to Medicare prematurely. For example, the third quartile of illness severity was 49% (95% CI, 45%-53%) more likely to switch relative to the quartile that included the patients least likely to be hospitalized within 12 months (Table 2).

**Table 1.** Association of Prematurely Switching Excess Months of Medicare and Additional Medicare Spending for Adults Aged 62 Years of Younger

Measure	Switch to Medicare		
	Premature^a^	At coordination period^b^	After coordination period or never^c^
Patients, No. (%)	37 696 (33)	30 190 (27)	45 807 (40)
Unadjusted measure			
Excess mo, mo/patient, mean (SD)	19 (12)	NA	NA
Total excess mo of Medicare spending	711 528	NA	NA
Medicare spending per patient, mean (SD), $	87 686 (104 196)	4176 (15 008)	3980 (18 862)
Total Medicare spending, $	3 305 404 749	126 063 115	182 302 583
Adjusted measure^d^			
Medicare spending, mean (95% CI), $	86 198 (85 176-87 219)	5198 (4968-5427)	4531 (4313-4749)
Absolute difference in mean Medicare spending, mean (95% CI), $^e^	0 [Reference]	81 000 (79 971-82 029)	81 667 (80 611-82 722)

^a^ Premature switches are defined as a switch from an employer-based group health plan to Medicare before the end of the coordination period.

^b^ Switches at the coordination period include switches between the end of the coordination period and 90 days after.

^c^ Switching after the coordination period entails switching more than 90 days after the coordination period or not switching at all.

^d^ Adjusted measures estimated using ordinary least squares, controlling for patient characteristics and comorbidities, facility characteristics, and zip code–sociodemographics (eTable in the Supplement).

^e^ The inverse of the coefficients for indicator variables designating whether a patient switched to Medicare regularly and whether a patient either switched to Medicare late or never.

**Table 2.** Association of Probability of Hospitalization and Likelihood of Prematurely Switching From an Employer-Based Health Plant to Medicare for Adults Aged 62 Years or Younger

Quartile^**a**^	Adults, 18-62 y (n = 113 693)	
	HR (95% CI)^b^	*P* value^**c**^
1, least likely to be hospitalized in 12 mo	1 [Reference]	NA
2	1.23 (1.20-1.27)	<.001
3	1.49 (1.45-1.53)	<.001
4, most likely to be hospitalized in 12 mo	1.71 (1.65-1.76)	<.001

Abbreviations: HR, hazard ratio; NA, not applicable.

^a^ Quartiles for 12-month probability of hospitalization. The probability of hospitalization among patients with an employer-based group health plan was estimated via a Cox proportional hazards model, modeling the association between premature switching from an employer-based group health plan to Medicare and probability of being hospitalized in 12 months. Estimated probabilities were obtained using multivariable logistic regression in patients with traditional Medicare.

^b^ An HR of greater than 1 signifies an increased likelihood of switching early.

^c^ Statistical significance was found at *P* < .001.

For adjusted measurements, patients who prematurely switched to Medicare from their EGHP cost Medicare $81 000 (95% CI, $79 971-$82 029) more than patients who switched at the coordination period and $81 667 (95% CI, $80 611-$82 722) more than patients who switched late or never (Table 1). From 2007 to 2017, premature switches cost Medicare an additional $3.05 billion (95% CI, $3.01-$3.09 billion).

## Discussion

Nearly one-third of adults 62 years or younger who began dialysis with an EGHP switched to Medicare prematurely, resulting in more than $3 billion of additional Medicare spending from 2007 to 2017. Patients who were more likely to be hospitalized were also more likely to switch from their EGHP to Medicare prematurely.

While premature switches increase Medicare spending, many are likely unavoidable because of unemployment associated with the start of dialysis. Blanket prohibitions on early switching would be costly for patients who are unemployed and require Consolidated Omnibus Budget Reconciliation Act, or COBRA, coverage.^3,4^ However, frequent premature switches to Medicare likely discourage EGHPs from funding cost-saving interventions that prevent ESKD in chronic kidney disease^5^ because switching to Medicare prematurely offloads more than $80 000 of financial risk per patient. More measured policies that share the risk of dialysis between EGHPs and Medicare, such as having Medicare pay EGHPs a capitated rate for premature switchers (similar in principle to Medicare Advantage), could incentivize EGHP investments aimed at preventing ESKD without increasing patients’ out-of-pocket spending.

This study was limited because of the inability to know why patients switched to Medicare or the employment status of the patients. Results were also subject to residual confounding and relied on the 2728 Form, which has incomplete comorbidity capture.^6^ Despite these limitations, there were large spending differences between premature switches to Medicare compared with switches to Medicare at or after the coordination period or never switching to Medicare. Policy makers should consider ways to share the financial risk of dialysis with EGHPs.

## Notes

Supplement. **eAppendix.** Supplementary Methods. **eTable.** List of Covariates for Regressions **eReferences.**
