# Supplementary material for: The Cost of Transferring Dialysis Care From the Employer-Based Market to Medicare
Source: JAMA Netw Open. 2021 Mar 18;4(3):e212113. doi: 10.1001/jamanetworkopen.2021.2113 (PMC7974636; doi:10.1001/jamanetworkopen.2021.2113)
Supplement: Supplement. — eAppendix. Supplementary Methods. eTable. List of Covariates for Regressions eReferences. [file jamanetwopen-e212113-s001.pdf]

Supplemental Online Content

Lin E. The cost of transferring dialysis care from the employer-based market to Medicare. *JAMA Netw Open*. 2021;4(3):e212113.
doi:10.1001/jamanetworkopen.2021.2113

eAppendix. Supplementary Methods
eTable. List of Covariates for Regressions
eReferences

This supplemental material has been provided by the authors to give readers additional information about their work.

© 2021 Lin E. *JAMA Network Open.*

eAppendix. Supplementary Methods

Data and Population

The University of Southern California's institutional review board approved this study. I followed the Strengthening the Reporting of Observational Studies in Epidemiology (STROBE) reporting guideline and conducted all analyses from June 2020 to January 2021.

I used data from the United States Renal Data System (USRDS), a national registry jointly sponsored by the National Institutes of Health (NIH) and the Centers for Medicare and Medicaid Services (CMS).[1] The USRDS is a deidentified registry of administrative data already collected by the Centers for Medicare & Medicaid Services, and thus patients do not provide informed consent. The dataset comprises all US patients with end-stage kidney disease (ESKD), irrespective of primary or secondary payer, linked to data from Medicare, including the enrollment file and a 100% sample of fee-for-service (FFS) Medicare claims. Thus, I was able to use the USRDS to longitudinally track patients' primary and secondary payers and FFS

Medicare spending (primary and secondary).

The USRDS also contains data from the CMS-Form 2728, which is submitted on all incident patients with ESKD within the first 45 days of initiating dialysis. Because these data are submitted for all patients, I was able to observe patient-level characteristics at the start of dialysis even for patients without Medicare. I obtained dialysis facility characteristics from CMS-Form 2744, an annual survey that is administered to dialysis facilities. Finally, I linked these data to the American Community Survey to obtain zip code level sociodemographics.[2]

My primary cohort was all adults aged 62 years or younger in the US initiating dialysis for ESKD between January 1, 2007, and December 31, 2014. I restricted my study population to patients with an employer-based group health plan (EGHP). To develop a model that estimated "sickness," I defined a similar (62 years or younger) comparison population among patients who had Medicare prior to starting dialysis. Because the last year of available follow-up for my dataset was 2017, I did not include patients initiating dialysis after 2014 to ensure that I could follow patients for the full coordination period. I excluded patients 63 and older in my primary analysis because they may be likely to retire during the EGHP coordination period. I expanded my sample to include all adults (ages 18 and older) in a sensitivity analysis. Sensitivity analyses did not change the direction of results, though the magnitude of spending estimates increased. Readers may obtain results from the sensitivity analyses on request.

Defining the Coordination Period and Assigning Premature Switches to Medicare

Although the coordination period is 30 months for all patients starting dialysis with an EGHP, the end of the period relative to the start of dialysis varies based on dialysis modality.[3] Patients using in-center hemodialysis become Medicare eligible on the first day of the fourth calendar month of dialysis. For patients with an EGHP, this marks the start of the coordination period, when Medicare becomes the secondary payer for 30 calendar months. Therefore, the coordination period for most patients with an EGHP lasts from the beginning of the fourth calendar month of dialysis until the last day of the 33rd calendar month of dialysis.

Patients with an EGHP who either start with home dialysis (home hemodialysis or peritoneal dialysis) or switch to home dialysis before the first day of the fourth calendar month become Medicare eligible on the first day of dialysis. The coordination period for these patients lasts from the first day of dialysis until the last day of the 30th calendar month.

© 2021 Lin E. *JAMA Network Open.*

Given these coverage rules, I defined a premature switch as a patient who obtained FFS Medicare prior to the end of the coordination period: the last day of the 33rd calendar month for patients using in-center hemodialysis and the last day of the 30th calendar month for patients using home dialysis before the fourth month. If a patient died prior to the end of the coordination period, I defined a premature switch as a patient who obtained FFS Medicare prior to death.

Because patients who switch at a standard time are likely different from patients who switch late or not at all, I divided the remaining patients into those who switched at a standard time (at the coordination period) and those who switched after the coordination period or never. I defined a standard switch as a patient who switched between the end of the coordination period and 90 days after the coordination period. I defined a late switch (ie, a switch after the coordination period) as a switch at least 91 days after the coordination period.

Medicare Spending During the Coordination Period

For each patient, I determined when the coordination period was supposed to end based on the type of dialysis used on the first day of the fourth calendar month. I defined Medicare spending during the coordination period as all Part A and B claims that Medicare paid, irrespective of whether Medicare was primary or secondary payer. I adjusted all spending for inflation to 2017 dollars.[4]

Covariates

I adjusted all models for patient (age, sex, race, ethnicity, employment status, primary cause of ESKD, whether the patient received nephrology care prior to dialysis, comorbidities, and body mass index), facility (whether the facility was free-standing or hospital-based, profit status, total number of patients, total number of patients receiving peritoneal dialysis, patient to nurse ratio, and patient to staff ratio), zip code sociodemographic (total population, median household income, percent of zip code below the poverty line, percent of zip code unemployed, percent of zip code without a high school degree, median rent, and whether the zip code was urban or rural), and temporal (month and year fixed effects) characteristics (**eTable 2** for full list). Patient race was obtained from the CMS-2728 Medical Evidence Form, which is submitted by dialysis facilities for all patients who received incident dialysis within 45 days of starting dialysis. Patient race and ethnicity are self-reported by patients at the time of filling out the form.

Statistical Analyses

I totaled the unadjusted number of premature Medicare switches and the total extra months of Medicare. To estimate whether patients with an EGHP that were more likely hospitalized were also more likely to prematurely switch, I assigned a risk score to each patient with an EGHP. I did this by using the probability of hospitalization as a proxy measure for sickness. First, from the comparison cohort of patients with FFS Medicare, I used a multivariable logistic regression model to estimate whether the patient was hospitalized within the first 12 months of dialysis with the above covariates as predictors. From these coefficients, I estimated the predicted probability that a patient with an EGHP would be hospitalized within the first 12 months of dialysis. By stratifying patients by quartile of predicted probability, I ranked patients by likelihood of hospitalization.

I chose to estimate a separate probability of hospitalization model instead of using previously constructed comorbidity scores, such as the Charlson Index or the Elixhauser Comorbidity Index. I did so for 2 reasons. First, outpatient dialysis costs tend to be fixed for all

© 2021 Lin E. *JAMA Network Open.*

patients. Much of the variation in costs (and a large overall share of spending) are attributable to hospitalizations and complications related to dialysis. Thus, if a health plan wanted to avoid covering expensive patients, they would probably start by identifying patients at high risk for hospitalization. And second, previously validated comorbidity scores are generally constructed using all patients and not just dialysis patients. To the extent that complications related to dialysis deviate from the general population, it is advantageous to re-estimate this model in the population of interest.

In patients with an EGHP, I estimated a Cox proportional hazards model to estimate time to obtaining FFS Medicare as primary payer as a function of the sickness quartile. This model estimates the relative change in likelihood of premature switching to Medicare by level of sickness.

I next compared total Medicare spending during the coordination period between those who switched prematurely and those who waited until the end of the coordination period to switch. Because patients who switch are on average different from patients who do not switch, I assessed differences in spending using ordinary least squares, adjusting for the above confounders. I used regression coefficients to estimate the marginal effect, or the average change in Medicare spending when a patient prematurely switches from an EGHP to Medicare as primary payer. All models used robust standard errors. My a priori significance level was 5%, and all hypothesis tests were 2-sided. I used SAS version 9.4 (SAS Institute) and Stata version 14.0, MP edition (StataCorp LLC).

© 2021 Lin E. *JAMA Network Open.*

eTable. List of Covariates for Regressions

**Patient level**	**Facility level**	**Zip code level**
Age<br>18-45<br>46-55<br>56-65<br>≥66 (for full population)	Type of facility<br>Freestanding<br>Hospital-based	Total population<br>0 - <5000<br>5000 - <15,000<br>15,000 - <25,000<br>25,000 - <50,000<br>≥50,000
Sex<br>Male<br>Female	Profit status	Median income, $<br>0 - <25,000<br>25,000 - <50,000<br>50,000 - <75,000<br>75,000 - <100,000<br>≥100,000
Race<br>White<br>Black<br>Asian<br>Other<br>Ethnicity<br>Hispanic	Total patients<br>1-50<br>51-100<br>101-150<br>≥151	% Below poverty line<br>0 - <15<br>15 - <25<br>≥25
Employment status<br>Unemployed<br>Employed<br>Retired (age)<br>Disabled<br>Other	Total peritoneal dialysis patients<br>0<br>1-10<br>11-25<br>26-50<br>≥50	Unemployed, %<br>0 - <5<br>5 - <10<br>10 - <15<br>≥15
Primary cause of ESKD<br>Diabetes<br>Hypertension<br>Other	Patient: nurse ratio<br>>0 – 10<br>>10 – 15<br>>15 – 20<br>≥20	Without high school diploma, %<br>0 - <15<br>15 - <30
Received Nephrology Care prior to ESKD	Patient: staff ratio<br>>0 – 4<br>>4 – 5<br>>5 – 6<br>>6 – 7<br>>7 – 8<br>8	Median rent ($)<br>0 - <750<br>750 - <1000<br>1000 - <1250<br>≥1250
Comorbidities<br>Alcohol dependence<br>Amputation<br>Atherosclerotic heart disease<br>Cancer<br>Congestive heart failure		

© 2021 Lin E. *JAMA Network Open.*

Chronic obstructive pulmonary disease<br>Prior stroke / transient ischemic attack<br>Diabetes:<br>Insulin dependent<br>No medications<br>Oral medications<br>Retinopathy<br>Drug dependence<br>Hypertension<br>Disability<br>Non-renal congenital abnormality<br>Other cardiac disease<br>Peripheral vascular disease<br>Smoker<br>Toxic nephropathy		
Body mass index, calculated as weight in kilograms divided by height in meters squared<br><18.5<br>18.5 - <30<br>30 < 40<br>≥40		

© 2021 Lin E. *JAMA Network Open.*

eReferences

1. United States Renal Data System. *2019 USRDS Annual Data Report: Epidemiology of Kidney Disease in the United States*. National Institutes of Health, National Institute of Diabetes and Digestive and Kidney Diseases; 2019. Accessed May 10, 2020. https://usrds.org/2019/view/Default.aspx
2. The United States Census Bureau. The American Community Survey. Published 2012. https://www.census.gov/programs-surveys/acs/
3. Centers for Medicare & Medicaid Services. Medicare Coverage of Kidney Dialysis and Kidney Transplant Services. Published July 2017. Accessed October 9, 2017. https://www.medicare.gov/Pubs/pdf/10128-Medicare-Coverage-ESRD.pdf
4. Bureau of Labor and Statistics. CPI Inflation Calculator. Accessed November 20, 2015. http://data.bls.gov/cgi-bin/cpicalc.pl

© 2021 Lin E. *JAMA Network Open.*
